# Systematic dissection of biases in whole-exome and whole-genome sequencing reveals major determinants of coding sequence coverage

**DOI:** 10.1038/s41598-020-59026-y

**Published:** 2020-02-06

**Authors:** Yury A. Barbitoff, Dmitrii E. Polev, Andrey S. Glotov, Elena A. Serebryakova, Irina V. Shcherbakova, Artem M. Kiselev, Anna A. Kostareva, Oleg S. Glotov, Alexander V. Predeus

**Affiliations:** 1 Bioinformatics Institute, Saint Petersburg, Russia; 2 Department of Genomic Medicine, D. O. Ott Research Institute of Obstetrics, Gynecology, and Reproduction, Saint Petersburg, Russia; 3 Department of Genetics and Biotechnology, Saint Petersburg State University, Saint Petersburg, Russia; 4 Cerbalab LTD, Saint Petersburg, Russia; 5 Institute of Translational Biomedicine, Saint Petersburg State University, Saint Petersburg, Russia; 6 City Hospital №40, Saint Petersburg, Russia; 7 Institute of Living Systems, Immanuel Kant Baltic Federal University, Kaliningrad, Russia; 8 Molecular Biology Division, Biomedical Center, LMU Munich, 82152 Planegg-Martinsried, Germany; 9 Almazov National Medical Research Centre, Saint Petersburg, Russia

**Keywords:** Medical genomics, Genetics research

## Abstract

Advantages and diagnostic effectiveness of the two most widely used resequencing approaches, whole exome (WES) and whole genome (WGS) sequencing, are often debated. WES dominated large-scale resequencing projects because of lower cost and easier data storage and processing. Rapid development of 3^rd^ generation sequencing methods and novel exome sequencing kits predicate the need for a robust statistical framework allowing informative and easy performance comparison of the emerging methods. In our study we developed a set of statistical tools to systematically assess coverage of coding regions provided by several modern WES platforms, as well as PCR-free WGS. We identified a substantial problem in most previously published comparisons which did not account for mappability limitations of short reads. Using regression analysis and simple machine learning, as well as several novel metrics of coverage evenness, we analyzed the contribution from the major determinants of CDS coverage. Contrary to a common view, most of the observed bias in modern WES stems from mappability limitations of short reads and exome probe design rather than sequence composition. We also identified the ~ 500 kb region of human exome that could not be effectively characterized using short read technology and should receive special attention during variant analysis. Using our novel metrics of sequencing coverage, we identified main determinants of WES and WGS performance. Overall, our study points out avenues for improvement of enrichment-based methods and development of novel approaches that would maximize variant discovery at optimal cost.

## Introduction

Next-generation sequencing (NGS) is rapidly becoming an invaluable tool in human genetics research and clinical diagnostics^1–3^. Practical use of NGS methods has dramatically increased with the development of targeted sequencing approaches, such as whole-exome sequencing (WES) or targeted sequencing of gene panels. WES emerged as an efficient alternative to whole-genome sequencing (WGS) due to both lower sequencing cost and simplification of variant analysis and data storage^4^. More than 80% of all variants reported in ClinVar, and more than 89% of variants reported to be pathogenic, come from the protein-coding part of the genome; this number increases to 99% when immediate CDS vicinity is included. Even allowing for the sampling bias, there is an overall agreement that most heritable diseases appear to be caused by alterations in the protein-coding regions of the genome. Given this, WES has dominated the projects characterizing human genome variation as well as clinical applications.

The pioneering 1000 Genomes project^5^ could not statistically characterize many of the rare variants critical to diagnostics of Mendelian disease due to a limited sample size. In an attempt to get a representative picture of protein-coding variation in human population, 6,500 WES samples were sequenced during ESP6500 project^6^. When a much larger reference set of 60,706 WES experiments was compiled and uniformly processed by the Exome Aggregation Consortium (ExAC)^7^, it dramatically increased the accuracy of allelic frequency (AF) estimation in general population. This led to a surprising conclusion that up to 90% of variants reported as causative for Mendelian disease in ClinVar database are observed too often in healthy controls to directly cause disease^7^. The number of available WES experiments is rapidly increasing, and the latest Genome Aggregation Database (gnomAD) collection includes 123,136 WES experiments alongside with 15,496 WGS. Such impressive number of profiled individuals allows a much more thorough look at human coding genome variation, leading to many useful applications such as estimation of selective pressure across protein-coding regions^8^.

Several published studies have concentrated on comparing the performance of different exome capture technologies, or comparison between WES and WGS. With the emergence of commercial exome kits, three major manufacturers - Agilent, Illumina, and Nimblegen (Roche) - have become popular among users, representing the majority of all published WES studies. Early comparative studies have focused on comparison of target intervals of various exome kits, and identified several important biases inherent to WES technology, such as coverage biases in regions with very high or low GC content^9–11^. A later study comprised most hybridization-based capture technologies available at the time^12^, and showed specific features of each of the four exome kits, including GC-content bias and differences in the distribution of coverage. Similar observations were made in one of the most recent comparative studies^13^. However, these and other earlier works on the topic included very limited number of samples, often with large variation of sequencing depth, which may have interfered with consistent platform comparison. Only one of the recent studies included larger amount of samples that allowed to identify tendencies in cross-sample coverage unevenness^14^.

It is often assumed that WGS offers more uniform coverage of CDS regions due to the nature of hybridization-based enrichment process used in WES. Such differences in coverage evenness increase the costs of effective per-base coverage in WES, questioning the overall benefit from using WES instead of WGS. Hence, the issue of WES/WGS comparison has been addressed by several studies that sought out optimal sequencing method to achieve maximum coverage of the protein-coding regions of the genome (listed in Supplementary Table S1). One of these included Agilent and Nimblegen (Roche) WES capture technologies, that were compared with the conventional WGS approach in terms of resulting coverage per sequencing read and the efficiency of clinically significant SNV detection^15^. Similarly to earlier studies^9,10^, it was found that WES achieves similar percentage of well-covered CDS bases only when the average coverage is 2–3 times higher, and with a substantial sequence bias. In several more recent studies, it was repeatedly stated that WGS provides more even and unbiased coverage of coding regions and generates more accurate variant calls^13,16,17^.

It is already well understood that long-read sequencing dramatically increases the power and accuracy of complex variant discovery in the human genome^18^. With rapid development of 3^rd^ generation sequencing technologies, long-read resequencing of human genomes becomes an attractive and increasingly realistic option. For example, the highest throughput Oxford Nanopore device, PromethION, is expected to generate 30x long-read coverage of human genome for less than $1000. A recent publication has highlighted limitations of short-read technologies, identifying “dark” regions in the protein-coding parts of the genome, including numerous disease-causing genes^19^. At the same time, it is unclear what combination of methods would allow the best effective coverage for regions of interest. There is a defined need for a robust statistical framework that would allow accurate evaluation of method performance on the level of coverage and before variant identification. Our study describes such framework, and uses it to find important determinants of coding sequence coverage in the human genome.

## Results

### Coverage efficiency analysis within and between CDS regions

**Figure 1:**
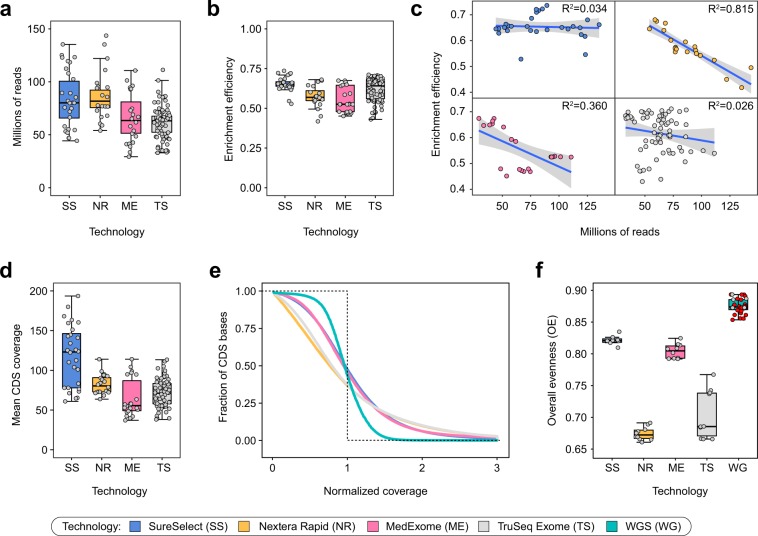
Coverage of target regions across WES and WGS samples. (**a,b,d**) Total read depth (**a**), target enrichment efficiency (**b**) and mean CDS coverage (**d**) for all samples for each platform. (**c**) A scatterplot of enrichment efficiency plotted against total read depth. Lines are linear regression fits with 95% confidence intervals indicated as grey envelopes. (**e**) The distribution of the normalized coverage for all WES technologies compared to WGS. Dotted line represents ideal case baseline, i.e. all bases covered at mean value. (**f**) Overall evenness (OE) scores for all four WES technologies and WGS. Red points indicate WGS samples obtained from open sources, while grey points represent our dataset. For plots (**e,f**) a subset of 10 samples with similar mean coverages was selected for all WES platforms.

We started off by characterizing the efficiency of CDS interval coverage by current WES and WGS technologies. It is important to note that most modern variant calling tools ignore reads with mapping quality (MQ) less than 10 and reads marked as PCR or optical duplicates; thus, such reads were removed when calculating coverage. All WES samples irrespective of the platform showed 50–70% efficiency of target enrichment and a similar distribution of sequencing depths across our WES dataset (Fig. 1a,b), corresponding to 38 ± 5 fold enrichment of target regions (Supplementary Fig. S1). Interestingly, we observed a weak trend showing that libraries having higher depth of sequencing tend to show less efficient exome enrichment. The strength of the trend depends on the particular technology: for SureSelect and TruSeq Exome kits the trend is almost absent (R^2^ = 0.034 and R^2^ = 0.026, respectively), while for MedExome and Nextera Rapid the dependence is much more pronounced (R^2^ = 0.360 and R^2^ = 0.815) (Fig. 1c).

Mean coverage of CDS regions in our dataset was comparable among different WES technologies (~ 70x), with exception for SureSelect, that had mean coverage of ~120x (Fig. 1d). We then calculated profiles of normalized coverage across CDS bases (Fig. 1e). In order to characterize the overall evenness of CDS coverage (OE), we have used the score developed by Mokry *et al*.^20^ (see Methods). Normalized coverage profiles and OE scores showed that both Illumina kits perform significantly worse than SureSelect and MedExome, while all exome platforms provided less even coverage than PCR-free WGS (Fig. 1e,f).

**Figure 2:**
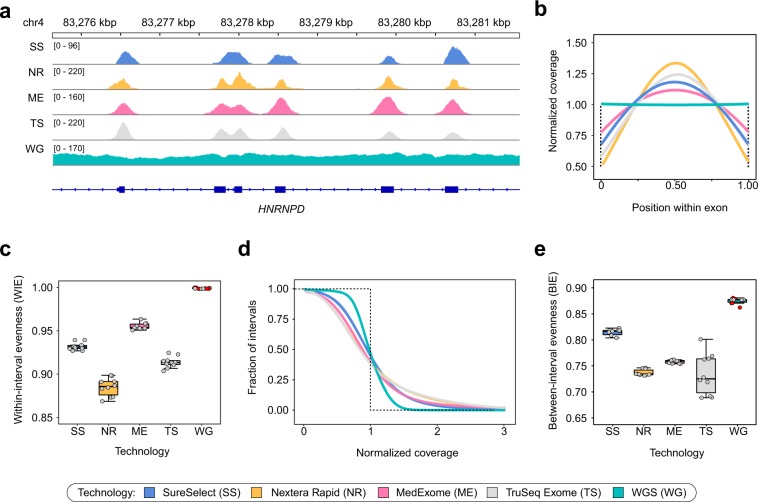
Different technologies exhibit specific patterns of coverage within exons and differ coverage distribution within exons. For all plots, a subset of samples was used as described earlier. (**a**) Example of sequencing coverage patterns across exons of the *HNRNPD* gene. Selected samples with similar mean CDS coverage are shown. (**b)** Distribution of relative coverage from the start to the end of target interval, averaged over all CDS regions. (**c**) Within-interval coverage evenness (WIE) values calculated from distributions shown in (**b**) (see Methods for more details; all capture technologies differ in pairwise U-test with Holm-Bonferroni FDR correction (adjusted p-value < 0.001)). (**d**) Distribution of normalized mean coverage across CDS intervals. As in Fig. 1, dotted line represents ideal case baseline. (**e**) Between-interval coverage evenness (BIE) values derived from normalized coverage curves shown in (**d**). Red points indicate WGS samples obtained from open sources, while grey points represent our dataset.

To dissect potential sources of coverage bias we defined two possible components of coverage evenness: coverage distribution between different CDS regions (between-interval evenness, BIE), and uniformity of coverage within individual intervals (within-interval evenness, WIE). The latter type of coverage unevenness is inherent to WES platforms; hence, we first questioned whether it explains the difference between WES and WGS in the OE scores. Indeed, visual inspection of coverage profiles on individual CDS regions suggests that exome platforms highly vary in WIE (Fig. 2a). To more accurately assess the observed differences, we calculated average profiles of relative coverages and WIE scores for all CDS regions (Fig. 2b). We found that WIE scores are well correlated with the OE (Fig. 2c), however, WIE does not completely explain differences observed in Fig. 1f. Similar results were obtained by calculation of WIE profiles across CDS intervals including flanking regions and with exon length stratification (Supplementary Fig. S2). As anticipated, WGS did not show any noticeable within-interval unevenness, confirming that such type of coverage bias is specific to exome sequencing. Since our results suggested WIE is not the only source of increased coverage bias in WES, we next calculated profiles of relative mean interval coverage across all CDS regions to estimate BIE (Fig. 2d). We observed that, while WGS generally performed better than WES, exome platforms showed a distinct pattern of between-group differences (Fig. 2e) that explains the discrepancy between OE and WIE scores, implying that the overall coverage evenness is the product of both BIE and WIE.

### Relative importance of coverage bias determinants in WES and WGS

**Figure 3:**
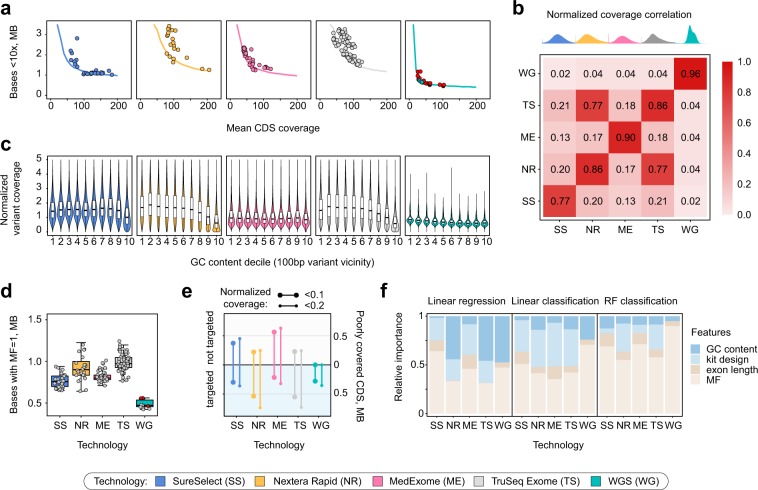
Modeling of CDS coverage identifies key determinants of coverage evenness. (**a**) A model based on normalized coverage patterns suggests existence of coverage limits for each technology. Solid lines correspond to model predictions of the amount of bases covered <10x depending on mean CDS coverage. Dots are samples analyzed in the study. (**b**) A heatmap showing average correlation between mean coverages for each exon. Distributions on top are distributions of per-interval normalized coverages. (**c**) GC-bias of coverage at variant sites with different GC-content of 100 bp vicinity (median GC-content in each bin: 0.33, 0.38, 0.42, 0.45, 0.49, 0.53, 0.57, 0.61, 0.65, 0.71). (**d**) Comparison of the amount of CDS bases covered only by multimapping reads for each technology. (**e**) Total length of targeted and not targeted CDS regions with reproducible low (<0.1 or <0.2 average) normalized coverage. (**f**) Relative importance of different exon features for prediction of exon coverage using linear regression (left), linear classification (middle), or random forest classification (right; see Methods for the details of importance calculation). Red points in panels (**a**,**d**) indicate WGS samples obtained from open sources, while grey points represent our dataset.

To more thoroughly characterize the capabilities and limitations of resequencing approaches, we have constructed a model to predict sequencing coverage of CDS regions that accounts for both between- and within-interval evenness (see Methods). Quite surprisingly, model-based prediction of the amount of bases covered at less that 10x at different mean coverages showed that all platforms, including WGS, have a certain amount of bases that are not covered at the required depth even at 200x average coverage (Fig. 3a). For common 30x WGS samples, 788 kbp of CDS sequences are covered less than 10x(with 407 kbp covered <10x at 200x mean coverage); for SureSelect, the best WES platform, 1180 kbp are predicted to have low coverage at 100x, and 970 kbp - at 200x). These results suggest that there are certain sources of reproducible coverage bias for both WES and WGS. We have set out to explore exactly how reproducible are these coverage biases, and what is the relative importance of different sequence features for the efficient coverage of CDS regions and variant discovery in exome sequencing.

We first evaluated the reproducibility of normalized coverage profiles for each technology. To this end, we estimated the correlation of per-interval normalized coverages across all samples for each technology. As seen from Fig. 3b, coverage bias in both WES and WGS has a systematic component. Among different WES platforms, SureSelect had the lowest reproducibility of coverage across CDS regions, and the two Illumina technologies had significant cross-correlation, suggesting that our estimates reflect specific features of capture process and bait design (Fig. 3b). Notably, WGS has shown higher correlation of normalized coverages (i.e., a more reproducible coverage bias) when compared to WES. To further validate this assumption, we selected 10 representative samples for each technology and evaluated the fraction of intervals with low (<0.1) normalized coverage in at least one sample (“union”) that also have low coverage in all 10 samples (“intersection”). As expected, such intersection-to-union ratio was highest for WGS and lowest for SureSelect (Supplementary Fig. S3).

We then turned to dissect specific covariates that affect CDS coverage in exome and genome sequencing. We first analyzed the variation in sequencing depth across regions with different GC-content, as GC-content has been referred as a major source of coverage bias in WES^9,13^. We calculated normalized coverage at ~180,000 ClinVar variant sites divided into 10 deciles dependent on GC-content of 100 bp variant vicinity, and found that both Nextera Rapid and TruSeq Exome capture kits performed worse than the others in GC-rich regions and better in the AT-rich ones (Fig. 3c). Among all four WES technologies, MedExome and SureSelect showed the best results with almost no dependence of read depth at variant site on the GC-content of the surrounding region. We also discovered a slight decrease in mean sequencing depth in GC-rich regions for WGS libraries. As WES platforms are assumed to perform worse in regions with extremely high or low GC-content, we also compared the distribution of normalized coverage for intervals with lowest (0–20%, 26 kbp) and highest (80–100%, 63 kbp) GC-content values. Our analysis showed that, in contrast to the results obtained using ClinVar variants, WES platforms perform worse than WGS in regions with extreme GC content (especially GC-rich regions, Wilcoxon test p-value < 0.001) (Supplementary Fig. S4). However, total length of regions with such extreme GC-content values is much smaller compared to the limits given by our coverage model (Fig. 3a). Given these results, we conclude that, despite high differences in coverage of GC-rich regions between WES and WGS, GC-bias is not a dominant factor of poor coverage for best WES platforms as well as WGS.

We then investigated another plausible source of coverage bias, namely, mappability limitations in short-read sequencing technologies. CDS regions are often considered unique and non-repetitive; though several examples of large repeated CDS elements have been noted^21^. Only recently the problem has received more focused attention^19^. Curiously, we noticed that for some genes there is a substantial decrease in read depth after exclusion of reads with low mapping quality (MQ). We conservatively defined multimapping fraction (MF) as the proportion of sequencing coverage that results from reads with MQ = 0. We then calculated MF for each exome base-pair and for individual CDS regions, and analyzed the amount of bases or intervals with high MF (for interval-level analyses, we focused on intervals with MF > 0.4, as this threshold generated 452 kb of sequences of interest, nearly matching the numbers observed in coverage model analysis (Fig. 3a)). On average, exome kits had more bases with higher MF (and, in particular, MF = 1, i.e. all coverage resulting from reads with zero mapping quality) than WGS (Supplementary Fig. S5, Fig. 3d). Strikingly, we found virtually no dependence of the amount of bases covered by multimapping reads on read length (Supplementary Fig. S6); and the difference between different WES platforms and WGS appears to be explained mostly by insert length of the sequenced fragment (Supplementary Fig. S7). In agreement with this hypothesis, Roche MedExome (WES platform with the largest insert) showed the smallest amount of bases covered by ambiguously mapped reads at all cutoffs (Supplementary Fig. S5). Overall, ~500 kbp of CDS sequences have MF = 1 even in WGS samples, suggesting that coverage limits for WGS arise mostly from mappability issues.

Finally, we questioned whether a substantial proportion of CDS regions with low normalized coverage in WES samples is simply not targeted by the capture probes. We first evaluated the intersection of target regions of each WES kit and CDS intervals (Supplementary Table S2). The results show that all platforms declare to cover most of CDS regions (with >90% CDS bases included in the bait intervals), with Illumina designs being the most comprehensive (99.1% of CDS bases). Interestingly, despite the high total length of declared SureSelect bait intervals (60.5 Mb vs. ~45 Mb), it targets the smallest fraction of CDS bases compared to other technologies, as well as the lowest number of ClinVar pathogenic variants (Supplementary Table S2). All kits included in the study do not feature extended UTR coverage, including only ~20% of GENCODE v19 UTR regions.

To assess whether the aforementioned bait design parameters introduce a significant coverage bias, we overlapped regions with normalized coverage significantly lower than 0.1 (or 0.2) (see Methods) with the bait design files for each WES technology, and calculated the fraction of poorly covered bases not overlapping target regions. Our analysis showed that for most platforms a large fraction of poorly covered bases falls into non-targeted regions of the exome (Fig. 3e). It is also apparent that best WES platforms (SureSelect and MedExome) are almost identical to WGS in the number of targeted bases that are poorly covered in all samples.

In order to compare the relative importance of different factors influencing CDS coverage, we fitted a linear regression model to predict normalized per-interval coverage depending on GC-content, interval length, multimapping fraction, and inclusion of the interval into the exome kit design. Analysis of the model showed that for SureSelect and Roche MedExome platforms multimapping fraction and inclusion are the most important predictors of normalized coverage, while GC-content is the major determinant of coverage for both Illumina kits. Importantly, the relative importance of GC-content was substantially decreased when using a linear classifier of poorly covered CDS regions (normalized coverage <0.1) (Fig. 3f). Even more pronounced reduction of GC content importance and increase of MF role was observed when we used random forest classifier to predict exons with low normalized coverage. Observed trend allows us to make an important conclusion: while sequence composition and kit design influence exon coverage in general, mappability limitations become a dominating factor of poor exon coverage (Fig. 3f).

**Figure 4:**
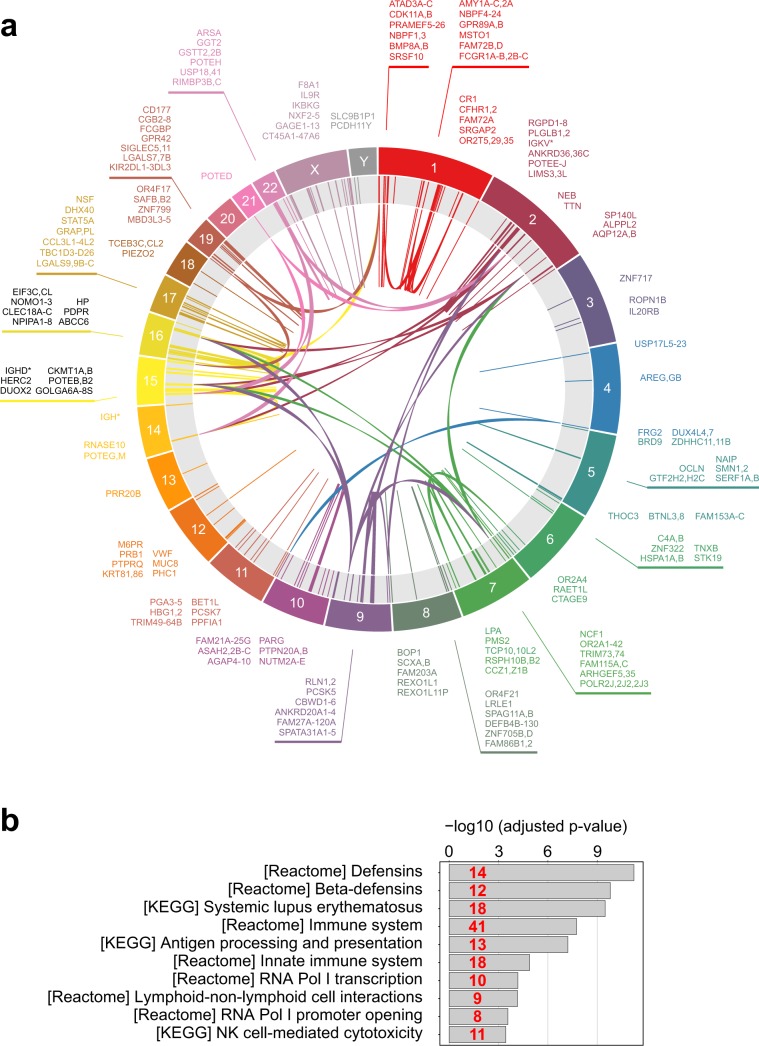
Summary of repetitive human CDS regions inaccessible by current WES and WGS technologies. (**a**) Circa diagram showing cross-mappability of CDS regions. Only a subset of clinically relevant genes is shown to decrease diagram complexity. (**b**) MsigDB enrichment analysis of genes with CDS regions having MF > 0.4 using canonical pathways (CP) list. Top-10 significant hits are shown. Numbers in red indicate the number of genes in each overlap.

Among regions with high MF, we found ~2000 exons corresponding to more than 500 genes, including known disease genes and cancer driver genes (Supplementary Table S3; Fig. 4a). Enrichment analysis of these genes over canonical pathway list from MSigDB showed significant overlaps with diverse immune system-related gene sets (Fig. 4b). This result is not surprising given that immunity-related genes are among the most duplicated gene families in the vertebrate genomes^22^. Our analysis only accounts for chromosomal parts of the 1000 Genomes assembly (also known as “b37”), which is most often used for variant calling. Including alternative contigs (totaling 3–4 Mbp depending on genome version and annotation, Supplementary Fig. S8) would certainly increase the ambiguity, especially when not using ALT-aware alignment and variant-calling tools. Importantly, current genome annotations also contain up to ~40 kbp of coding sequence in primary extrachromosomal scaffolds, which are not covered by any of the current WES platforms.

### Variant calling performance on WES and WGS data

**Figure 5:**
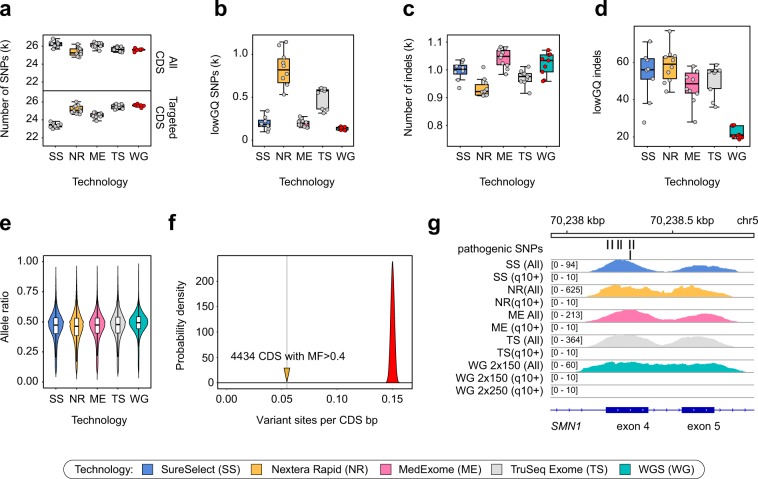
Variant calling biases of 4 exome capture technologies and WGS. (**a**) Total number of variants detected inside GENCODE v19 coding sequences (all CDS) and within targeted CDS regions (targeted CDS). (**b**) Number of variants with low genotype quality (lowGQ) according to the GATK GenotypeRefinement annotation. (**c,d**) Number of all called (**c**) and low-genotype quality (**d**) indels. (**e**) Allele ratios at heterozygous variant sites. (**f**) Per-nucleotide density of ExAC variant sites in regions with high fraction of multimapping read coverage (solid line) compared to the distribution of expected variant site density calculated from random subsets of CDS regions (see Methods for details). (**g**) Example of an unmappable CDS region in the exons 4–5 of the *SMN1* gene containing several well-established pathogenic variants. Two coverage tracks (including reads with low MQ and excluding these reads) are shown for each technology.

In order to see how the observed coverage limitations translate into our ability to detect variation, we have compared the number of variants discovered within CDS for each of the samples in our dataset to summarize the performance of resequencing technologies. We found that for all platforms the numbers of discovered in-CDS variants is approximately the same (Fig. 5a, upper panel), while the number of variants inside CDS that fall within targeted regions is in good correlation with the overall size of the CDS regions covered by each design (Fig. 5a, lower panel). The amount of variants with low genotype quality was significantly higher for both Illumina technologies and the highest for the Nextera Rapid kit, while best exome platforms did not differ from WGS in variant call quality (Fig. 5b). Similar results were observed for small insertion-deletion variants (indels); however, WGS have generated slightly fewer lowGQ variants than any of the WES platforms (Fig. 5c,d). Overall, it is very important to note that restriction of variant calling to the bait regions decreases the power of variant discovery in WES, which is otherwise comparable to that of WGS.

Allele bias is often considered one of the major determinants of poor variant quality in WES samples. To address this issue, we then assessed the allele ratios at heterozygous variant sites. We found no difference between allele ratio distributions in these samples (Fig. 5e) though for Nextera Rapid the distribution is more heavily-tailed with more variant sites having greater coverage of the reference allele. We also calculated the allele bias (AB) ratio characterizing the median amount of reads supporting reference allele in heterozygous variant sites. The AB estimate was found to be ~0.53 for MedExome, SureSelect, and TruSeq, while for Nextera Rapid the number was somewhat greater (~0.55).

We also statistically assessed the effect of mappability limitations on variant discovery. To this end we first calculated variant site density (VSD) (based on ExAC dataset) for each b37 CDS region and assessed the relationship between such variant site density and MF. We observed that regions with higher values of MF (~0.4 and higher) contain fewer variant sites per base-pair compared to the rest of the exome (Supplementary Fig. S9). To evaluate the degree of this difference, we calculated mean VSD in CDS regions with MF > 0.4, as well as similar value for 100000 sampled sets of 4434 CDS regions with MF < 0.4. This analysis showed a dramatic decrease in mean VSD for coding regions with high fraction of non-uniquely mapped reads (Fig. 5f). Similar results were observed when comparing variant site counts using a set of 10 representative samples for each platform studied in this work (Supplementary Fig. S10). This result confirms that mappability is an important determinant of sequencing coverage that substantially affects variant discovery. A profound example of nearly unmappable CDS regions with high clinical relevance are the *SMN1* and *SMN2* genes, mutations in which cause spinal muscular atrophy (SMA) - a fatal neurological disorder with an early age of onset. Indeed, we found that for seven out of eight exons (harboring several well-established pathogenic variants, e.g. rs104893934, rs397514518, rs104893933) inside SMA genes all coverage results solely from reads with zero mapping quality in both WES and WGS (Fig. 5g) (including 2 × 250 bp WGS). Consistently with these observations, no variants are characterized in these regions in gnomAD exomes and genomes (http://gnomad.broadinstitute.org/gene/ENSG00000172062).

## Discussion

Despite the ready availability of NGS methods, modern large-scale sequencing projects studying human rare diseases and population-scale variation are facing a difficult choice. The often heated “WGS vs. WES” debate is complicated by difficulty of estimation of indirect costs of each method. Most sources agree that WGS is 2 to 3 times more expensive, but the number changes a lot between different centers. However, perhaps more importantly, there are few criteria useful to compare method efficiency. The power of each method could be indirectly evaluated using percentage of successfully diagnosed cases for Mendelian diseases; most such studies report modest improvement in diagnostic rates when using WGS over WES^23^. Other studies have aimed to compare the performance of different WES kits with each other and with WGS more directly, using coverage and variant identification statistics. However, due to the constant improvement in exome kit design and standardization of variant calling procedures, these studies quickly become outdated. Furthermore, low number of reported samples have hampered the use of advanced statistical approaches that would allow to carefully address sample-to-sample variation necessary for such comparison. In this study we have leveraged a unique exome and genome dataset in an effort to provide a universal framework for an unbiased evaluation of modern WES and WGS.

We have found that while all WES technologies provide reasonable enrichment efficiencies, modern SureSelect and MedExome platforms offer substantially more even coverage than both solutions by Illumina (Fig. 1). It has been reported previously that WES provides much less even coverage than WGS^15,24^. Our results confirm these statements (Fig. 1e,f); however, a more careful look at different sources of coverage unevenness suggests that, at least in part, this difference results from within-interval unevenness (Fig. 2) that can be mitigated by increasing sequencing depth. Importantly, modelling of coverage distribution shows that all platforms (and both WES and WGS) have significant amounts of CDS bases that are effectively not covered at any sequencing depth (i.e., at least 407 kb for WGS and 960 kb for best WES; Fig. 3a). This result contradicts intuitive expectation of PCR-free WGS to uniformly cover all of the genome at least to some extent. The reason for such discrepancy is explained by exclusion of reads with zero mapping quality from our analysis pipeline. Variant calling software does not consider reads with low mapping quality; hence, such reads should be omitted in coverage analysis.

Mappability limitations of short reads render 478 ± 37 kb (for WGS) and 751 ± 34 kb (for best WES) of CDS regions unreachable for sequencing technologies. The problem of low-mappability regions is known; for some of the genes with poor mappability, complex statistical methods have been proposed to determine genotype likelihoods^21^. However, the mappability issue is often overlooked or considered insignificant for coding regions despite the fact that numerous clinically relevant regions are effectively unmappable (Fig. 4a, Supplementary Table S3), including well-characterized Mendelian disease genes (e.g., *SMN1/SMN2*, Fig. 5g). Statistical analysis of relative predictor importance suggests that, contrary to popular belief, mappability (and, for some kits, bait design) are the most important determinants of low coverage in WES samples. On the other hand, GC-content, which is usually considered as a major source of coverage bias^9,13,17^, virtually does not affect coverage for well-designed WES kits or PCR-free WGS (Fig. 3f).

It is important to note that variant calling for WES samples should not be restricted to targeted intervals and should rather include targeted intervals, CDS regions, UTR sequences and bases flanking CDS to improve the power of variant discovery (Fig. 5a,b). Overall, our modeling suggests that a WES sample sequenced with a common 100x average depth will provide significantly poorer coverage of only ~400 kb of CDS compared to a common 30x WGS sample, i.e. in ~1% of coding regions. These predictions are in good concordance with our analysis of variant calling results inside CDS regions (Fig. 5). Best WES platforms are virtually indistinguishable from WGS in both overall number of in-CDS variants discovered and fraction of low genotype quality variants, with WGS showing slightly better performance only for indels. Despite the fact these numbers do not directly estimate each technology’s sensitivity and specificity, they reflect absence of noticeable systematic differences between WES and WGS. A big limitation of all short-read sequencing technologies is their inability to accurately characterize complex structural variants, the problem which will only be solved with newer sequencing approaches based on long reads.

A recent review by Wright *et al*.^23^ suggested that WGS is more efficient than WES only by 2% of diagnosis rates on aggregate. Our observations suggest that WGS allows for more efficient coverage of only 1% of exome compared to best WES platforms, complementing the fact that only a small fraction of reported ClinVar pathogenic variants are not targeted by exome kits. Moderate rates of NGS-based diagnostics of monogenic diseases are likely explained by the lack of biological understanding of variant pathogenicity^25^. This, in turn, diminishes the role of technical WGS benefits, such as ability to identify numerous regulatory variants in intronic and intergenic regions. In many cases, annual re-analysis of undiagnosed samples using new biological data improves diagnosis rate^26^. Value of WGS will undoubtedly increase with better understanding of human genome regulation.

Several lines of evidence indicate that modern WES remains an excellent alternative to WGS in research and clinical applications. Moreover, current WES technologies can be further improved in several ways: first, support for longer insert sizes would decrease the impact of both mappability and WIE; second, inclusion of all currently annotated CDS regions to make coverage more comprehensive; and third, better probe design and improvement of hybridization process would alleviate remaining unevenness resulting from GC-content or other sequence-based determinants. In fact, the most recent WES solutions (e.g., produced by Illumina in conjunction with IDT) are reported to perform substantially better than NR or TS kits analyzed in this work, making WES samples approach WGS in terms of coverage distribution and eventually minimizing the diagnostic gap between WES and WGS approaches. When 3^rd^ generation methods become more widely adopted, one could envisage combinatorial methods that would benefit from long-read power of complex variant discovery, combined with high accuracy and cost efficiency of WES.

## Methods

### Sample collection

Peripheral venous blood samples were collected in EDTA from 167 patients with endocrine diseases, hereditary connective tissue disorders, orphan diseases and individuals from the control group. DNA was extracted with QIAsymphony automated station for the isolation of nucleic acids and proteins. The study was approved by the Review Board of Saint-Petersburg City Hospital No. 40 (Protocol 119, 09.02.2017) and Biobank of Center for Preventive Medicine (Protocol No. 02-05/15, 10.03.2015, and No. 05-05/15, 09.06.2015), Moscow. All patients gave informed consent for blood sampling, research, processing of personal data and storage of biological materials before collecting the samples and processing the medical history data. The study was performed in accordance with the Declaration of Helsinki.

### Exome library preparation

After DNA extraction, we prepared whole exome libraries with Illumina Nextera Rapid Capture Exome (24 samples), Nimblegen (Roche) SeqCap EZ MedExome (43 samples), Illumina Truseq Exome (72 samples), and Agilent Sureselect XT2 V6 technologies (28 samples).

### SeqCap EZ MedExome Kit (Roche, USA)

1 µg of human DNA in 1x Low TE buffer (pH = 8.0) was used as a starting material and sheared on Diagenode BioRuptor UCD-200 DNA Fragmentation System to the average DNA fragment size of 170–180 bp. The shearing conditions were as follows: L-mode, 50 minutes of sonication cycles consisting of 30 s sonication and 30 s pause. Library preparation and exome capture were performed using SeqCap EZ MedExome Kit (Roche, USA) following the SeqCap EZ Library SR User’s Guide, v5.1 without modification. DNA libraries were amplified using 7 PCR cycles, and 14 PCR cycles were performed for amplification of enriched libraries. Library quality was evaluated using QIAxcel DNA High Resolution Kit on QIAxcel Advanced System.

### Nextera® rapid capture exome kit (Illumina Inc., USA)

Library preparation and exome capture were performed following the Nextera Rapid Capture Enrichment guide v. 15037436 (Illumina Inc., USA) without modifications. 50 ng DNA was used as a starting material and 10 cycles of PCR were performed for pre-enrichment and post-enrichment PCR steps. Library quality was evaluated using QIAxcel DNA High Resolution Kit on QIAxcel Advanced System.

### TruSeq Exome Library Prep Kit (Illumina Inc., USA)

300 ng of human DNA in 100 μl of 1x TE buffer (pH = 8.0) was used as a starting material and sheared on Diagenode BioRuptor UCD-200 DNA Fragmentation System to the average DNA fragment size of 200 bp. The shearing conditions were as follows: L-mode, 45 minutes of sonication cycles consisting of 30 seconds sonication and 30 seconds pause. Shearing results were evaluated using QIAxcel DNA High Resolution Kit on QIAxcel Advanced System. Several (1–10) additional sonication cycles were performed to reach the desired 200 bp DNA fragment size peak, when needed. 100 ng of sheared DNA was used as a starting material for library preparation. Library preparation and exome capture were performed using TruSeq Exome Library Prep Kit following the standard TruSeq Exome Library Prep Reference Guide (Illumina Document # 15059911 v01). Library quality was evaluated using QIAxcel DNA High Resolution Kit on QIAxcel Advanced System.

### Agilent SureSelect XT2 Library Prep Kit ILM v.6

2 µg of human DNA in 100 μl of 1x Low TE buffer (pH = 8.0) was used as a starting material and sheared on Diagenode BioRuptor UCD-200 DNA Fragmentation System to the average DNA fragment size of 150–200 bp. The shearing conditions were as follows: L-mode, 60 minutes of sonication cycles consisting of 30 seconds sonication and 30 seconds pause. Shearing results were evaluated using QIAxcel DNA High Resolution Kit on QIAxcel Advanced System. Library preparation and exome capture were performed following the SureSelectXT Target Enrichment System for Illumina Multiplexed Sequencing Protocol (Version B5, June 2016) for 3 µg of starting DNA. Library quality was evaluated using QIAxcel DNA High Resolution Kit on QIAxcel Advanced System.

### Whole-exome sequencing

Illumina HiSeq 2500 and Illumina HiSeq. 4000 platforms were used for sequencing. Each exome library was sequenced using 101 bp (HiSeq 2500) or 150 bp (HiSeq 4000) paired-end reads. All WES samples satisfied the ExAC criterion of minimum 80% of CDS bases with 20x coverage.

### Whole-genome sequencing

For comparison of exome capture technologies with conventional WGS approach, we used several recent samples sequenced at Biobank genome facility^27^. WGS libraries were prepared using TruSeq DNA PCR-Free LT Library Prep Kit (Illumina, USA) according to the manufacturer’s protocol. Additionally we used PCR-free WGS data of the Genome In A Bottle (GIAB) consortium^28^ (Chinese and Ashkenazi trios), as well as several samples publicly available at the NCBI Sequencing Read Archive (SRA) (SRA IDs SRR2098244, SRR2969967, ERR2186302, SRX2798634, SRX2798624). For GIAB samples, we used pre-calculated Novoalign BAM files available at the GIAB FTP site (ftp://ftp-trace.ncbi.nlm.nih.gov/giab/ftp/data/). For our own WGS samples and samples downloaded from SRA, we used bwa mem v0.7.1 for read alignment. All BAM files were narrowed down to the GENCODE v19 CDS regions using bedtools^29^. We further downsampled the 300x BAM file for GIAB sample HG001 to obtain 5 separate BAM files with 60x mean coverage or 10 BAM files with 30x mean coverage (Fig. 5).

### Whole-exome sequencing data analysis

For all exome and genome samples, bioinformatic analysis of sequencing data was done using a pipeline based on bwa mem^30^, PicardTools v2.2.2 (http://broadinstitute.github.io/picard/) and Genome Analysis ToolKit (according to the GATK Best Practices workflow^31,32^). Sample genotyping was done in a cohort calling mode using GATK HaplotypeCaller. Variant calling was restricted to either bait regions for each technology or the CDS regions (see Results). Variants were filtered using Variant Quality Score Recalibration (SNV sensitivity 99.9%, indel sensitivity 90.0%). Annotation and subsequent filtration of variants was done using SnpEff and SnpSift tools followed by automated correction of reference minor alleles by RMA Hunter^33^. Hybrid selection metrics were calculated using CollectHsMetrics tool in the PicardTools package. Alignment data visualization was carried out in the Integrated Genomics Viewer (IGV)^34^.

### Interval file comparison

To analyze the proportion of CDS and UTR sequences covered by each technology’s declared design file, we used the bedtools package^29^. Reference GENCODE v19 genome annotation (http://gencodegenes.org/)^35^ was used for these estimations. Only chromosome located CDS regions of protein-coding genes were used in the analysis. We also used ClinVar database of variants implicated in human disease (build 2018-04-01) to assess coverage of important variant sites^36^.

### Coverage calculation

Modern best practices advise using GATK toolset for variant calling, which ignores reads with mapping quality (MQ) less than 10 and reads mapped as duplicate by Picard MarkDuplicates utility. Thus, all coverage calculations were done on BAM files with duplicate reads and reads with MQ < 10 removed. Exact coverage calculation pipeline is available at https://github.com/bioinf/weswgs. We also calculated multimapping fraction (MF) for each sample and for each CDS region by subtracting mean coverage after filtering by mapping quality (MQ > 10) from mean coverage before such filtering.

### Calculation of coverage evenness statistics

To analyze the distribution of coverage across target regions, as well as between-interval evenness (BIE) and within-interval evenness (WIE, or coverage smoothness), we used a combination of bedtools package and custom scripts in bash and Python (available at https://github.com/bioinf/weswgs). To collect normalized coverage profiles for each platform, BAM files were converted to a bedgraph format using bedtools. Next, the bedgraph file was intersected with the CDS regions according to GENCODE v19 genome annotation or the declared target regions for each technology. To calculate the coverage evenness and profiles of per-base normalized coverage we used the resulting bedgraph files to obtain fractions of bases having normalized coverage of at least N with N ranging from 0 to 3 with step 0.01. Overall evenness score was calculated as described^20^.

To calculate the between-interval evenness (BIE), mean sequencing depth was calculated for each interval. These coverage values were then processed similarly to per-base coverages. Between-interval evenness (BIE) measure was calculated similarly to the OE from the profiles of normalized mean coverages of individual intervals.

For calculation of the within-exon coverage distribution, all intervals having an average coverage of more than 10x in a sample were then divided into 100 bins of equal bp length. We then calculated normalized (divided by the mean coverage of a fragment) coverage in each bin. Then, mean coverage at each bin across all of the intervals was calculated for each sample. Within-interval evenness (WIE, or smoothness) was defined as the area under within-interval normalized coverage curve (restricted to the maximum value of 1)

$$WIE=\mathop{\sum }\limits_{i\,=0}^{100}(0.01\times min({x}_{i},1)),$$

where *i* is the bin number (relative distance within the interval with step of 0.01), and *x*_*i*_ is the normalized coverage in this bin.

### Variant calling performance analysis

To calculate the allele ratio distribution and the distribution of total and low genotype quality (lowGQ) variants, we used the VCF file resulting from the cohort genotyping of samples, and scripts written in Python (available at https://github.com/bioinf/weswgs). To calculate the mean coverage of variant sites depending on the GC-content of variant site neighborhood we selected 180452 known variants from the ClinVar database of clinically significant variants, and divided these variant sites into 10 equal groups depending on the GC-content (calculated by bedtools nuc) of the region 50 bp up- and downstream of the variant. We then calculated the read depth at all resulting variant sites using bedtools multicov.

### Modelling and investigating coverage biases

To construct a model of per-interval normalized coverage, we have calculated mean normalized coverage (*M*) of each individual CDS region in all samples sequenced with a particular technology, as well as the standard deviation (*s*) of this mean. We next predicted the amount of base-pairs with low (<10x) coverage (shown in Fig. 3a) in the following way: for each CDS interval *i* we sampled normalized coverage value *C*_*i*_ from normal distribution with mean *M*_*i*_ and standard deviation *s*_*i*_:

$${C}_{i}\sim N({M}_{i},\,{s}_{i}^{2})$$

We then calculating per-base normalized coverage by multiplication of *C*_*i*_ and a WIE profile for a given interval *i*. To do so, for each base pair *j* in interval *i* we calculated normalized coverage value *C*_*ij*_ as follows:

$${C}_{ij}={C}_{i}\times WI{E}_{ij},$$

where *WIE*_*ij*_ is the within-interval normalized coverage for base pair j (in other words, the expected coverage depth relative to the mean coverage of the region *i*). The resulting normalized coverage of each base-pair *ij* were used to calculate absolute read depth (*D*_*ij*_) at position *j* in interval *i*:

$${D}_{ij}={C}_{ij}\times D,$$

where D is the average read depth at targeted exome regions. The total number of bases covered with less than 10 reads (*D*_*ij*_ < 10) was then calculated for a range of exome average depths *D* = [20; 200].

To obtain a list of intervals that systematically have poor sequencing coverage for each technology (shown in Fig. 3e), we statistically evaluated the difference between the distribution of normalized coverage of each CDS region (*M*_*i*,_, *s*_*i*_) and the threshold value (0.1 or 0.2) using one-sample *t*-test with Holm-Bonferroni FDR correction.

To assess the reproducibility of coverage biases, we calculated mean pairwise correlation of vectors of per-interval normalized coverages across all samples sequenced with a particular platform (or all platforms in the study). Additionally, we selected a set of 10 samples for each technology and calculated the total length of intervals that have low (<0.1) normalized coverage in (a) any of the selected samples (“union”); and (b) all of the selected samples (“intersection”). We then calculated the intersection-to-union ratio (R) as the measure of coverage reproducibility:

$$R={L}_{intersection}/{L}_{union}$$

To assess the relative importance of different variables for prediction of normalized per-interval coverage, we used several machine learning models for both regression and classification tasks. For regression, we fitted a simple linear model describing mean per-interval coverage depending on GC-content, interval length, multimapping fraction, and a binary variable indicating inclusion of the interval into the exome design. For linear classification, we trained a logistic regression-based classifier to predict a binary variable indicating a normalized coverage <0.1 using the same variables as predictors. Similar setup was used for random forest (RF) classifier. All machine learning-based analyses were done using the ‘caret’ package^37^. Model testing was performed using 5-fold cross validation, accuracy and kappa-statistic values were used to select the best model. For RF classification, a model with mtry = 2 was selected. Tuning was performed using the default number of trees in the ensemble. For all models, predictor importance was analyzed using the default measures for linear and RF models provided in the ‘caret’ package.

### Estimation of the ExAC variant site density

Exome Aggregation Consortium (ExAC) variant calls (v. 0.3.1)^7^ were used to make statistical assessment of variant density. Each exome interval was annotated with the number of ExAC variant sites that fall inside this interval using bedtools intersect. Next, variant counts were transformed to per-nucleotide variant site density, and the resulting dataset was used for sampling procedures.

### Data availability and scripts

All statistical analyses were carried out using R v3.6. All machine learning analyses were done using caret v6.0-84. All graphs were plotted using ggplot2^38^ v3.2.1 and cowplot v1 packages. Scripts for data analysis and figures, as well as the processed data, can be found at https://github.com/bioinf/weswgs.

## Supplementary information

Supplementary Figures S1-S10.

Supplementary Table S1.

Supplementary Table S2.

Supplementary Table S3.
