# Supplementary material for: Systematic dissection of biases in whole-exome and whole-genome sequencing reveals major determinants of coding sequence coverage
Source: Sci Rep. 2020 Feb 6;10:2057. doi: 10.1038/s41598-020-59026-y (PMC7005158; doi:10.1038/s41598-020-59026-y)
Supplement: Supplementary file 1 — Supplementary Figures S1-S10. [file 41598_2020_59026_MOESM1_ESM.pdf]

Systematic dissection of biases in whole-exome and whole-genome sequencing reveals major determinants of coding sequence coverage

Yury A. Barbitoff[1,2,3,4], Dmitrii E. Polev[4], Andrey S. Glotov[2,5,6,7], Elena A. Serebryakova[2], Irina V. Shcherbakova[2], Artem M. Kiselev[8], Anna A. Kostareva[8], Oleg S. Glotov[2,6] , and Alexander V. Predeus[1]*

[1]Bioinformatics Institute, Saint Petersburg, Russia,
[2]Department of Genomic Medicine, D. O. Ott Research Institute of Obstetrics, Gynecology, and Reproduction, Saint Petersburg, Russia,
[3]Department of Genetics and Biotechnology, Saint Petersburg State University, Saint Petersburg, Russia,
[4]Cerbalab LTD, Saint Petersburg, Russia,
[5]Institute of Translational Biomedicine, Saint Petersburg State University, Saint Petersburg, Russia,
[6]City Hospital №40, Saint Petersburg, Russia,
[7]Institute of Living Systems, Immanuel Kant Baltic Federal University, Kaliningrad, Russia,
[8]Almazov National Medical Research Centre, Saint Petersburg, Russia.

*To whom correspondence should be addressed: predeus@bioinf.me, Bioinformatics Institute, Kantermirovskaya st. 2A, Saint Petersburg, 197342, Russia.

Table of contents

Supplementary Figures 2

Supplementary Figures

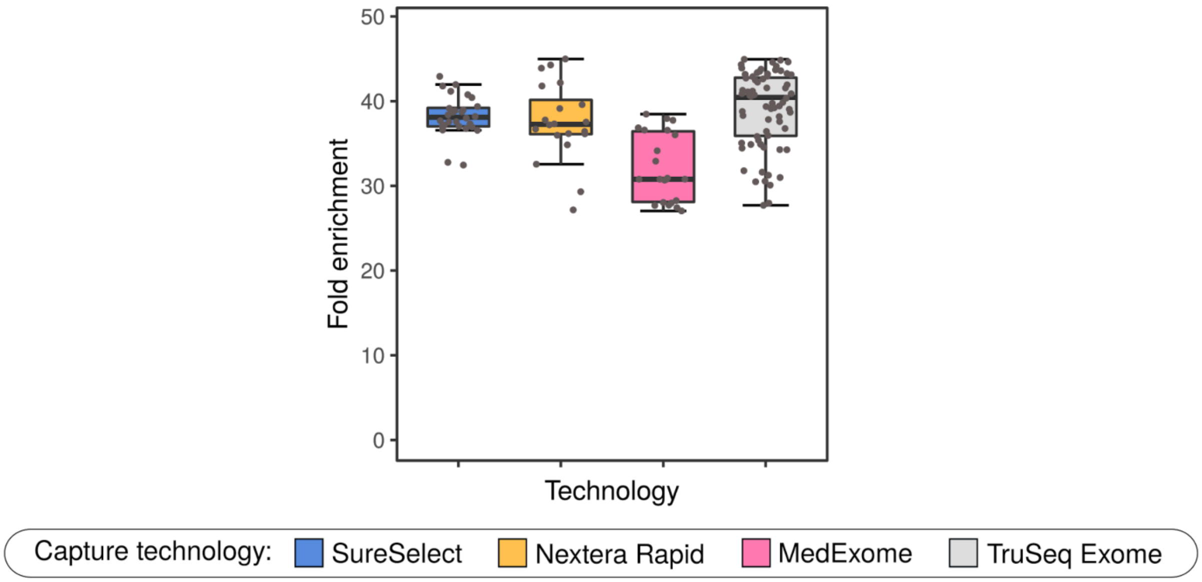

Supplementary Figure S1. Fold enrichment of CDS regions for WES samples included in the study.

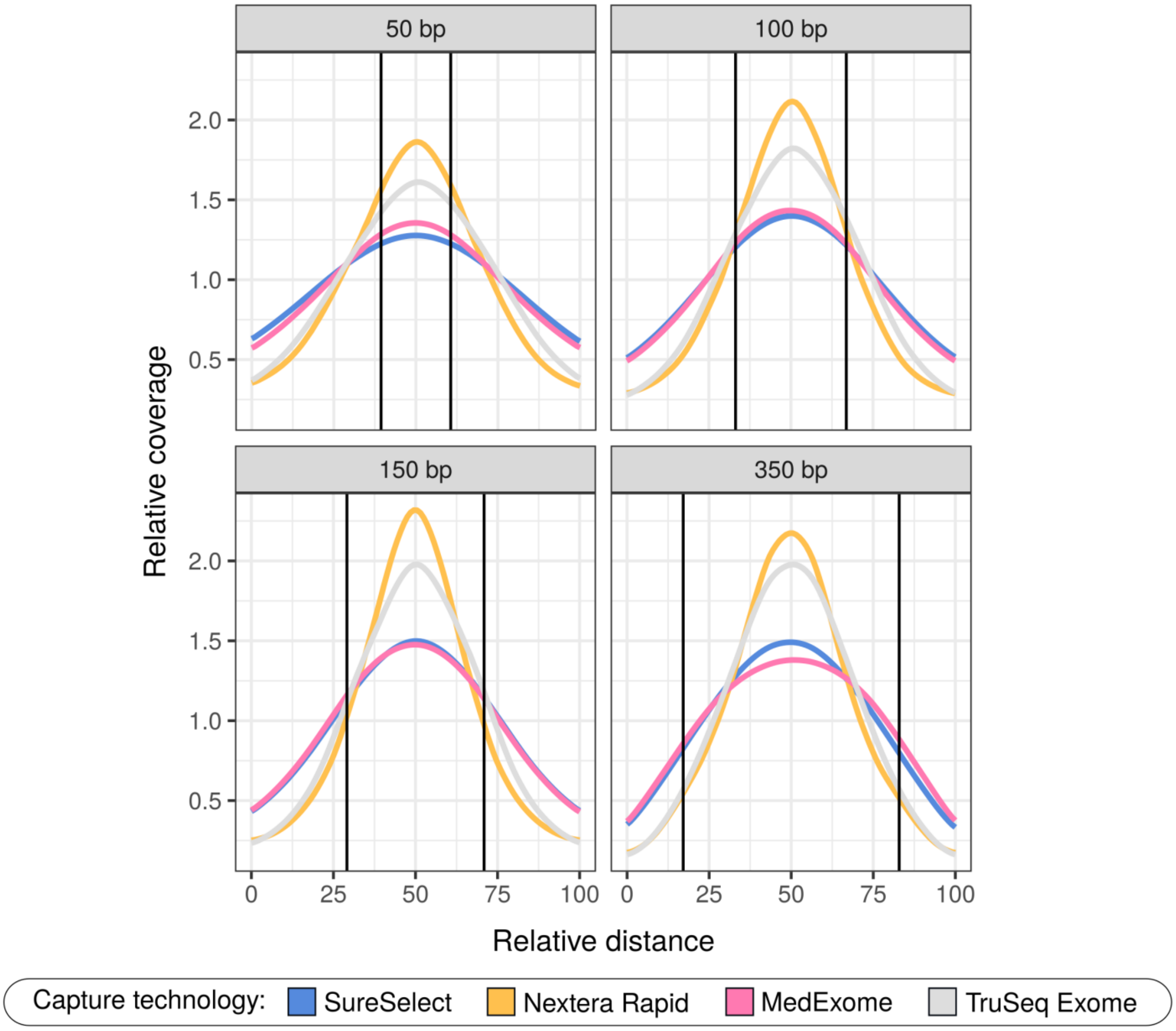

Supplementary Figure S2. Profiles of relative coverage within exons divided into 4 quartiles according to the length of an interval. 100 bp of flanking bases are included; solid lines delineate CDS margins.

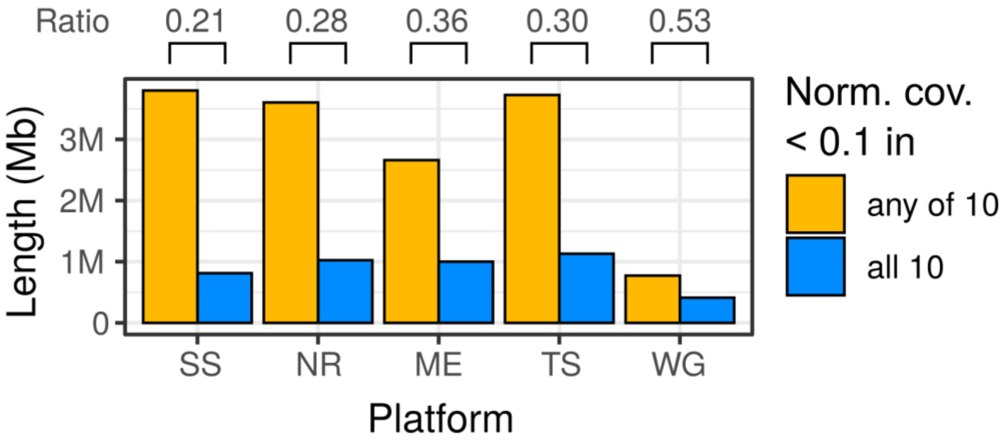

Supplementary Figure S3. Comparison of the total length of CDS regions that have low (< 0.1) normalized coverage in a set of 10 representative samples for each platform. Orange bars correspond to total length of regions that have low normalized coverage in at least 1 of 10 samples ("union"); blue bars correspond to regions that have low normalized coverage in all 10 samples ("intersection"). Numbers above the plot correspond to the ration of intersection to the union lengths.

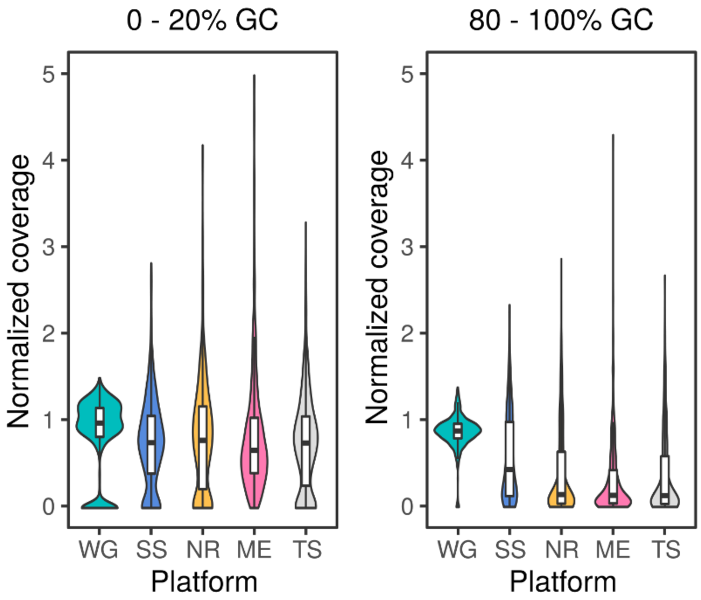

Supplementary Figure S4. Normalized coverage distribution for CDS regions with lowest (0 - 20%, left) and highest (80 - 100%, right) GC content. Only targeted by design regions are analyzed for WES platforms.

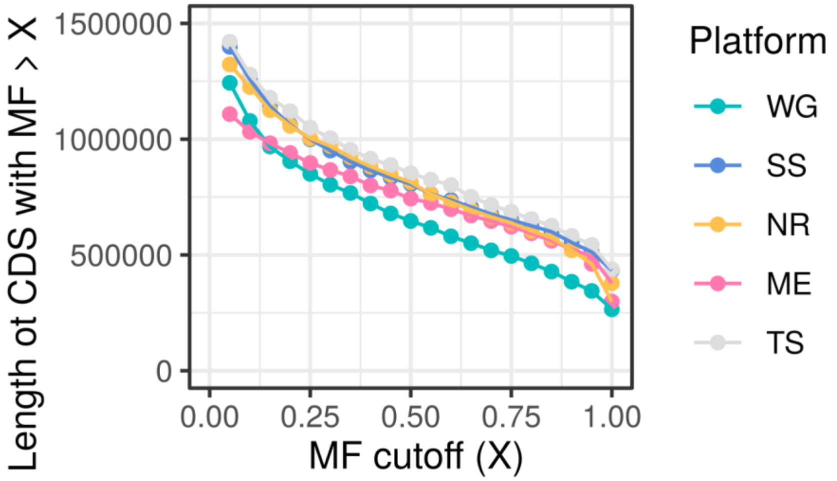

Supplementary Figure S5. The relationship between MF cutoff (X) and the total length of CDS intervals having MF > X. Note that Roche MedExome shows best performance among WES platforms.

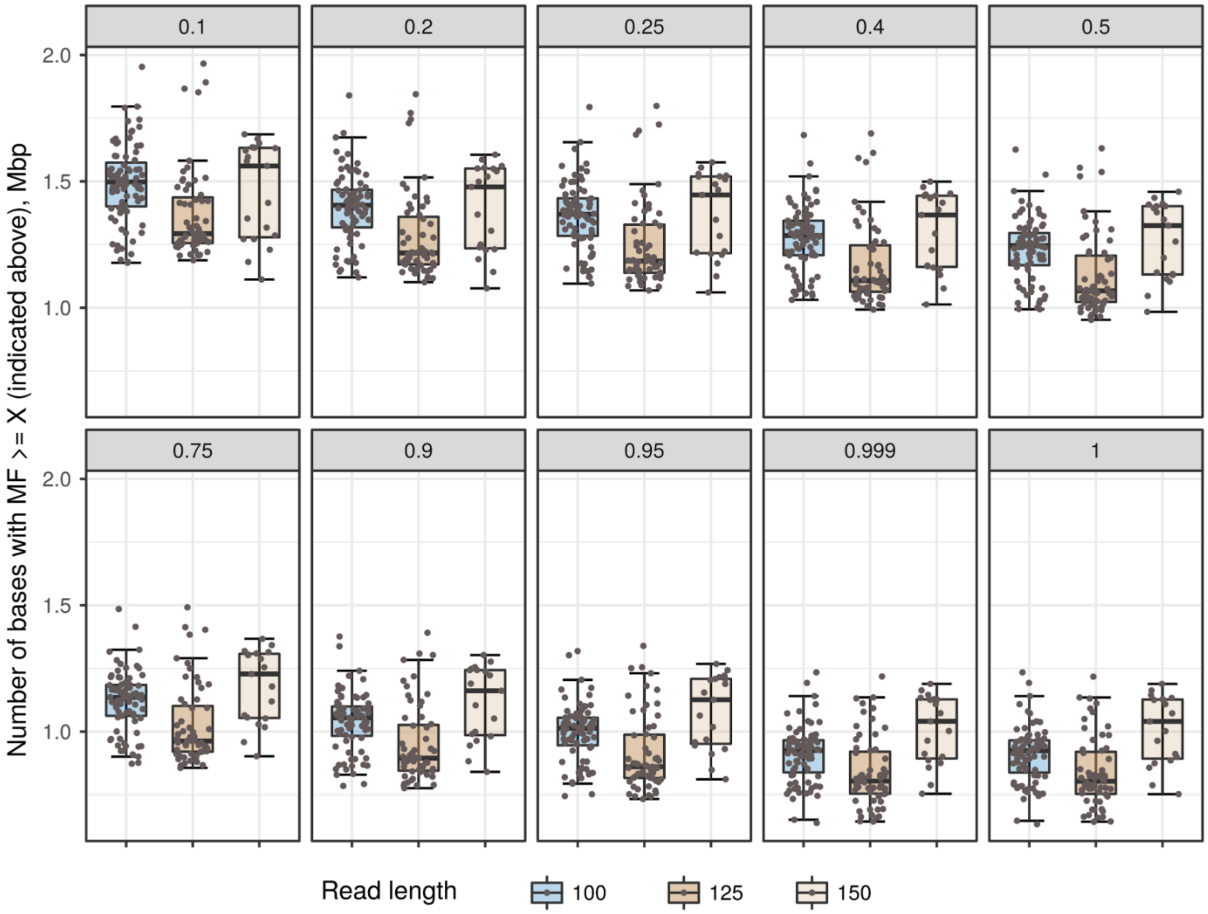

Supplementary Figure S6. The number of bases having an MF >= X (indicated above each plot) for WES and WGS samples having indicated paired end read lengths.

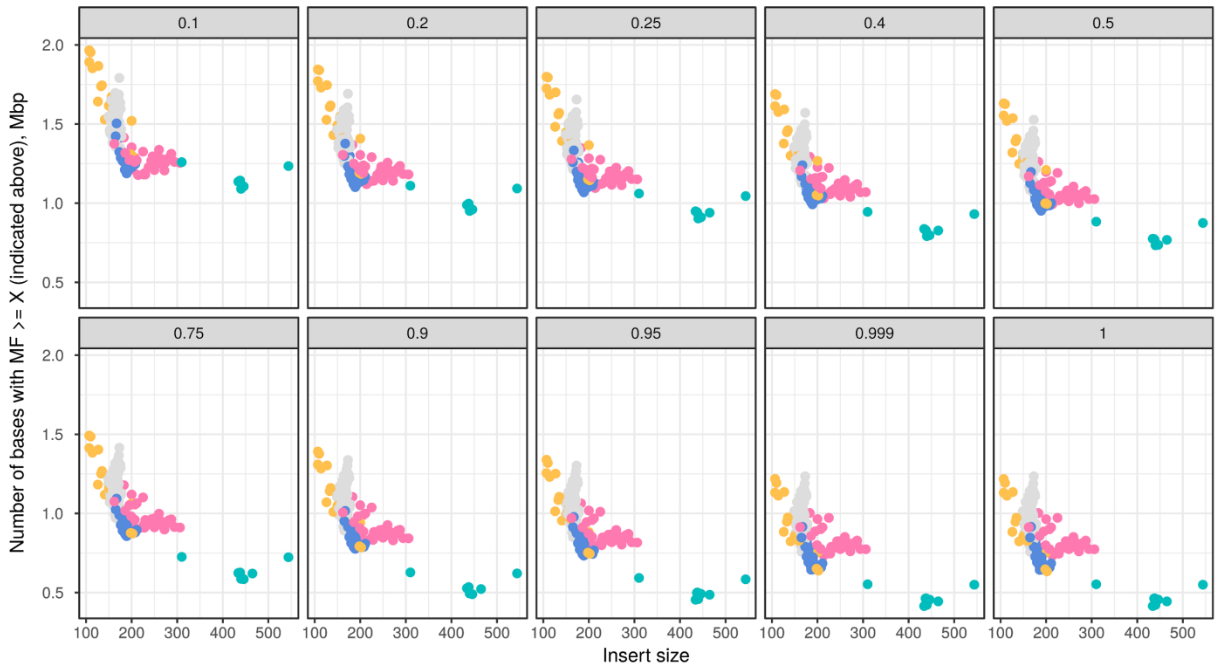

Supplementary Figure S7. The number of bases having an MF = X (indicated above each plot) for all samples plotted against the library mean insert size.

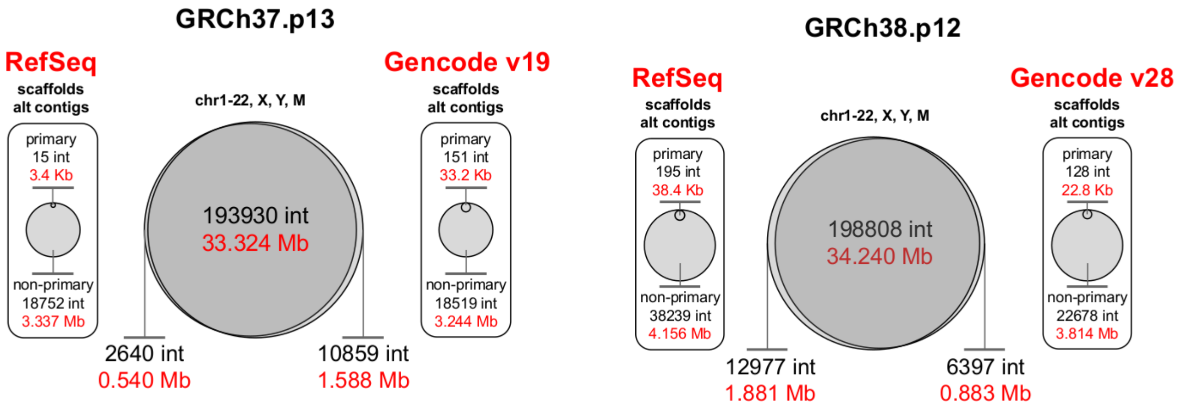

Supplementary Figure S8. Comparison of modern GRCh37- (left) and GRCh38-based (right) genome annotations by RefSeq and GENCODE. Middle, diagrams showing the overall agreement between annotations on chromosomal coding sequences. Summary of extrachromosomal coding sequences for each annotation source is shown aside of the central diagram.

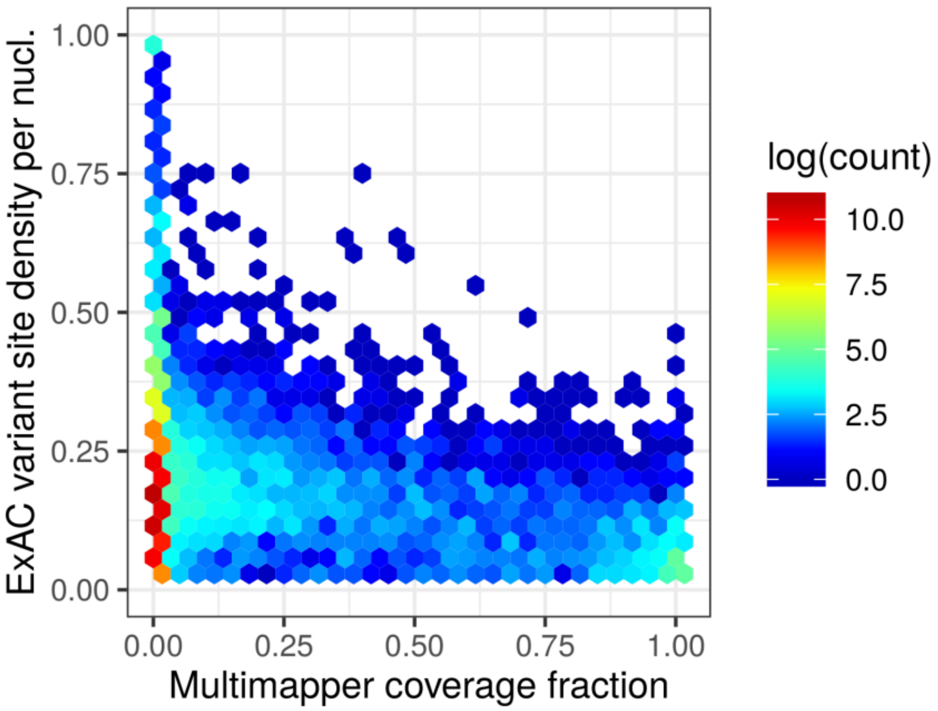

Supplementary Figure S9. The relationship between multimapper coverage fraction (MF) with the ExAC variant site density per nucleotide of CDS sequence. Hexagon color is proportional to the log10 of variant count in each bin.

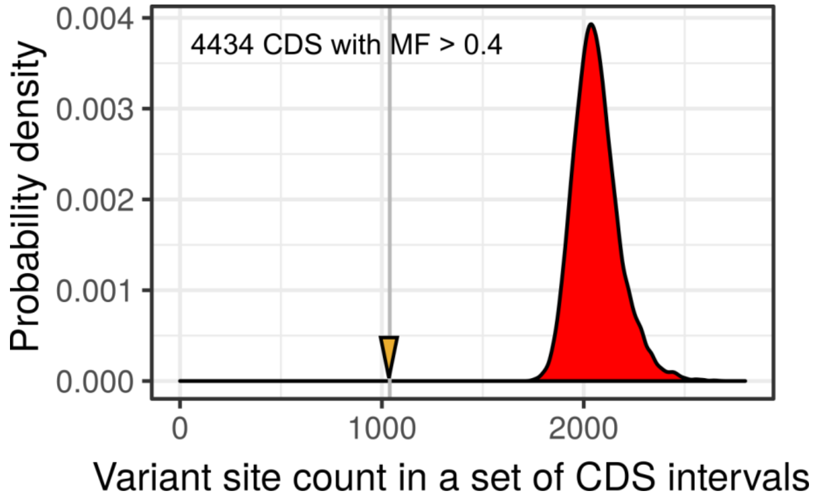

Supplementary Figure S10. The distribution of the expected variant site count in the 50 representative samples used for variant call analysis (Figure 5) in regions with MF < 0.4 (red density) compared to the observed number of variant sites identified within 4434 CDS regions with MF > 0.4 (yellow arrowhead).
